# Effects of Cyclization on Activity and Stability of α-Conotoxin TxIB

**DOI:** 10.3390/md18040180

**Published:** 2020-03-29

**Authors:** Xincan Li, Shuai Wang, Xiaopeng Zhu, Dongting Zhangsun, Yong Wu, Sulan Luo

**Affiliations:** 1 Key Laboratory of Tropical Biological Resources of Ministry of Education, Key Laboratory for Marine Drugs of Haikou, School of Life and Pharmaceutical Sciences, Hainan University, Haikou 570228, China; xincanll@163.com (X.L.); shuaiwang0927@163.com (S.W.); biozxp@163.com (X.Z.); zhangsundt@163.com (D.Z.); 2 Medical School, Guangxi University, Nanning 530004, China

**Keywords:** α-conotoxin TxIB, α6/α3β2β3 nAChRs, cyclization, activity, stability

## Abstract

α-Conotoxin TxIB specifically blocked α6/α3β2β3 acetylcholine receptors (nAChRs), and it could be a potential probe for studying addiction and other diseases related to α6/α3β2β3 nAChRs. However, as a peptide, TxIB may suffer from low stability, short half-life, and poor bioavailability. In this study, cyclization of TxIB was used to improve its stability. Four cyclic mutants of TxIB (cTxIB) were synthesized, and the inhibition of these analogues on α6/α3β2β3 nAChRs as well as their stability in human serum were measured. All cyclized analogues had similar activity compared to wild-type TxIB, which indicated that backbone cyclization of TxIB had no significant effect on its activity. Cyclization of TxIB with a seven-residue linker improved its stability significantly in human serum. Besides this, the results showed that cyclization maintained the activity of α-conotoxin TxIB, which is conducive to its future application.

## 1. Introduction

Nicotine acetylcholine receptors (nAChRs) are ligand-gated ion channels, which are key targets for the treatment of depression, addiction, Parkinson’s disease, neuralgia, Alzheimer’s disease, and cancer [1,2,3,4,5]. α6/α3β2β3 (α6β2*) nAChRs are highly expressed in the region of midbrain dopamine (DA) neurons in the central nervous system, which regulates the release of dopamine [6]. Several previous studies implied that α6β2* nAChRs were closely related to several neuropsychiatric disorders, such as Parkinson’s disease and nicotine addiction [7,8,9].

α-Conotoxins, which were discovered from *Conus* venom, are known as competitive antagonists for nAChRs. Some may have therapeutic potential [10,11,12,13,14]. Typically, α-conotoxins consist of 12–20 amino acids, with four cysteines resulting in two loops, and they are classified based on their various loop sizes [15,16]. Theoretically, four Cys residues can form three possible disulfide linkages, forming three isomers, including globular (CysI-CysIII, CysII-CysIV), ribbon (CysI-CysIV, CysII-CysIII), and bead (CysI-CysII, CysIII-CysIV) isomers [17]. In our lab, a novel α-conotoxin TxIB (α-CTx TxIB) was discovered from *Conus textile* [18]. Its globular isomer specifically inhibits rat α6β2* nAChRs with an IC_50_ of 28 nM but has no obvious effect on other subtypes of nAChRs. However, the bead and ribbon forms of TxIB are inactive for α6β2* nAChRs [19]. Therefore, the globular TxIB could be developed as a probe for studying the function and structure of α6β2* nAChRs, as well as neurological disorders, for instance, nicotine addiction and Parkinson’s disease.

Though α-conotoxins have been considered as useful pharmacological tools and drug leads, they also confront the generic problems of peptides being easily hydrolyzed by proteases, as well as having a short half-life and low bioavailability in vivo, thus affecting their pharmaceutical potential [20,21,22]. Therefore, several chemical modifications have been approached to ameliorate the metabolic stability of conotoxins, including cyclization, disulfide bond engineering, residue substitutions, N-terminal acetylation, glycosylation, PEGylation, etc. [23,24,25,26,27,28,29]. Among these strategies, the head-to-tail cyclization method was widely adopted to increase the ability of the peptide to resist enzymatic degradation, due to the inability of the enzymes to access the N and C termini of peptides [30]. Furthermore, the cyclic conotoxins also have greater conformational restrictions than their linear counterparts [31]. Several conotoxins, including χ-CTx MrIA, α-CTx MII, ImI, Vc1.1, RgIA, AuIB, and GeXIVA, have been successfully cyclized, and their stability has been improved to varying degrees [32,33,34,35,36,37,38]. According to these studies, cyclic conotoxins were regulated by the properties of the linker sequences, including the length and amino acid composition [39]. An appropriate linker length contributes to preservation of the original structure of conotoxins, while the amino acid composition may affect their activities. 

**Figure 1:**
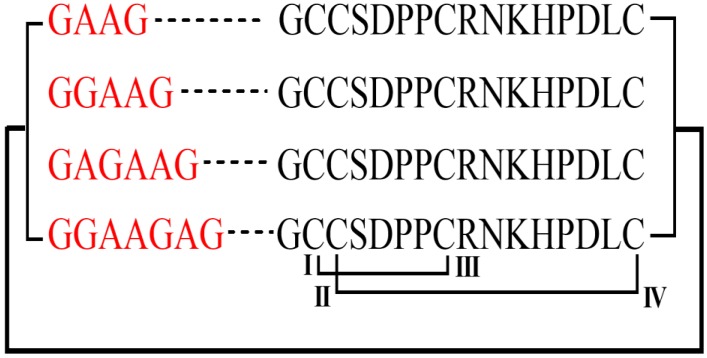
Amino acid sequences of cTxIB-4, 5, 6, and 7. The red letters indicate the four different linkers that were used to cyclize TxIB. The black bracket and connected line show the connection of the N-terminal and C-terminal of TxIB. The labeled black lines denote the disulfide connectivity.

In this research work, a cyclization strategy was selected to ameliorate the stability of TxIB in human serum while preserving its biological activity for rat α6β2* nAChRs. Therefore, four TxIB analogues were redesigned with four to seven residues in the linker region based on the distance between the N- and C-termini of TxIB (Figure 1). These mutants were synthesized using Fmoc-based solid-phase peptide synthesis (SPPS) on the 2-chlorotrityl chloride resin. Next, the effects of head-to-tail cyclization were evaluated for activity and stability. Compared to native TxIB, the four cyclic analogues still reserved similar activity. In addition, the stability of the analogue cTxIB-7 was improved significantly. The results have proved again that N- and C-terminal cyclization is an effective strategy to enhance the metabolic stability of conopeptides. 

## 2. Results

### 2.1. Synthesis, Cyclization, and Oxidative Folding of TxIB (cTxIB)

**Figure 2:**
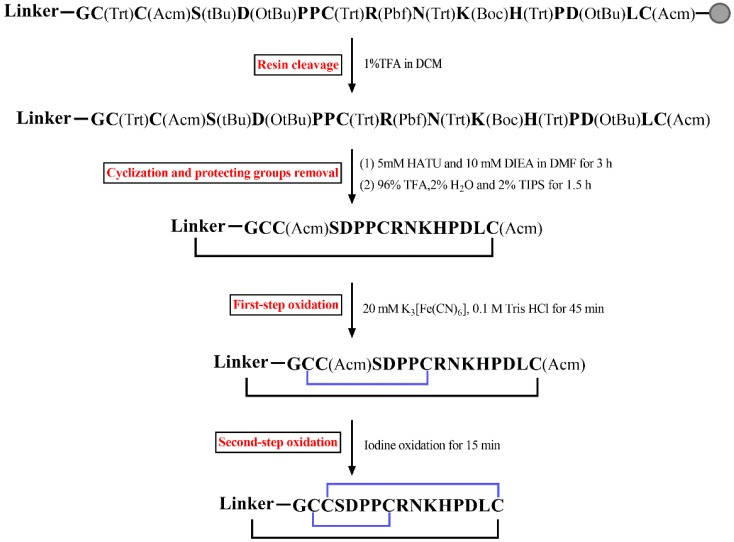
Schematic of the synthetic route for cyclic analogues of α-conotoxin TxIB.

All cyclic mutants were synthesized using standard SPPS on the acid-sensitive resin 2-chlorotrityl chloride, and the specific synthetic procedure of these peptides is shown in Figure 2. Firstly, linear peptides were produced using the standard Fmoc-SPPS (solid-phase peptide synthesis) method. After that, the resin was cleaved by 1% TFA. Then, these linear peptides were cyclized using HATU and DIEA in DMF. In this step, the reaction time was optimized to 3 and 6 h. The test results displayed that the effect of cyclization in 3 h was better than that in 6 h (Supplementary Material Figure S1). After 6 h of cyclization, more chemical by-products were produced, and it was difficult to separate the target peak using preparative HPLC. In contrast, the target peak was easily detected after 3 h of cyclization and it could be separated well. 

**Figure 3:**
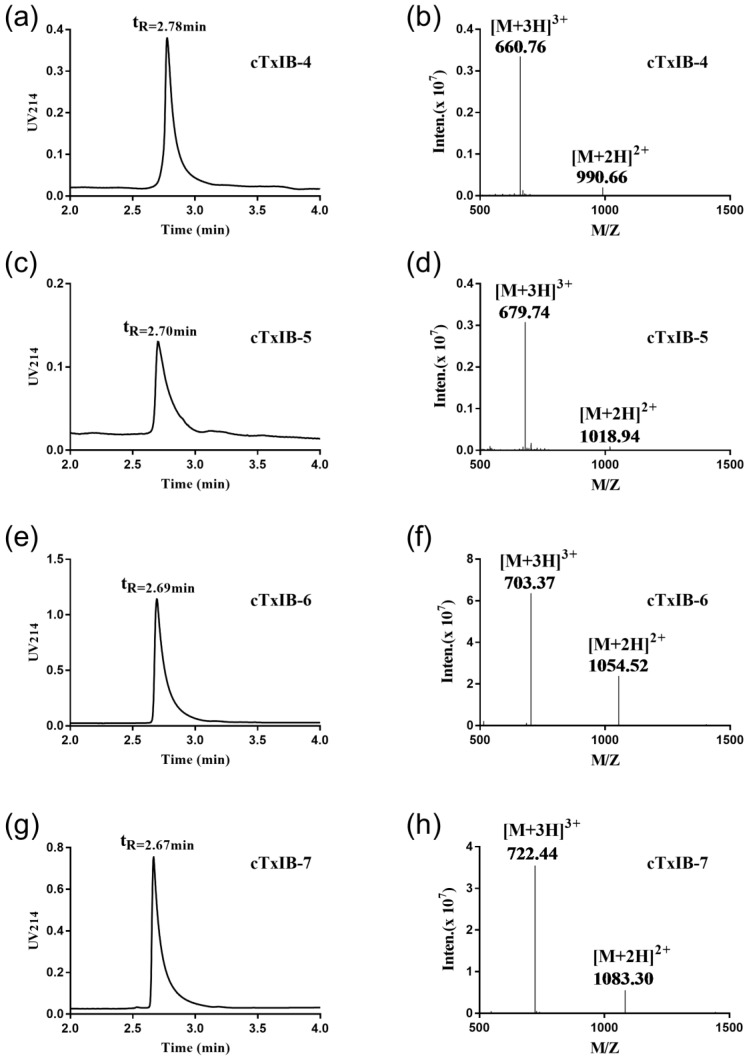
RP-UPLC and mass spectrometry analysis of the final cTxIB-4, 5, 6, and 7. (**a**) RP-UPLC chromatogram of cTxIB-4 with a retention time of 2.78 min; (**b**) ESI-MS data of cTxIB-4 with a mass of 1979.28 Da; (**c**) RP-UPLC chromatogram of cTxIB-5 with a retention time of 2.70 min; (**d**) ESI-MS data of cTxIB-5 with a mass of 2036.22 Da; (**e**) RP-UPLC chromatogram of cTxIB-6 with a retention time of 2.69 min; (**f**) ESI-MS data of cTxIB-6 with a mass of 2107.11 Da; (**g**) RP-UPLC chromatogram of cTxIB-7 with a retention time of 2.67 min; (**h**) ESI-MS data of cTxIB-7 with a mass of 2164.32 Da.

Then two pairs of disulfide bonds of cyclic analogues were connected by a two-step oxidation method. The first disulfide bond was formed by treatment with 20 mM K_3_[Fe(CN)_6_], and the second disulfide bond was produced by iodine oxidation. Finally, the purity of all cyclic peptides was determined using RP-UPLC, and the molecular weights of these peptides were detected by ESI-MS (Figure 3). Table 1 summarizes the theoretical and observed molecular weights of the intermediate and final products.

### 2.2. Electrophysiological Activity Measurements

**Figure 4:**
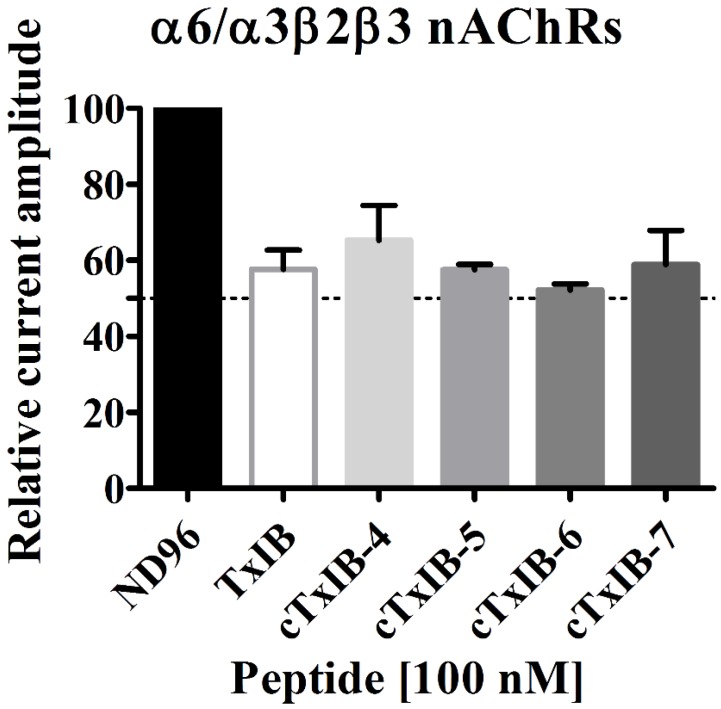
The relative current amplitude of TxIB and cyclized analogues at a concentration of 100 nM on rat α6/α3β2β3 nAChRs. The ND-96 solution was used as the negative control. Data points are mean ± SEM (n = 3–4). Statistical analysis was according to one-way ANOVA; * *p* < 0.05, ** *p* < 0.01, and *** *p* < 0.001 versus TxIB.

To determine whether the activity of TxIB would be affected after cyclization, the activities of four cyclic TxIB analogues were tested at α6β2* nAChRs expressed in *Xenopus* oocytes using electrophysiological assays. All analogues were screened at a single concentration of 100 nM, which was close to the IC_50_ of native α-conotoxin TxIB. The ND-96 solution was used as the negative control. The rα6β2* ACh-evoked current amplitude mediated by the inhibition of the peptides is shown in Figure 4. Relative to 42% inhibition of α6β2* nAChRs by TxIB, the inhibition of cTxIB-4, cTxIB-5, cTxIB-6, and cTxIB-7 were about 35%, 42%, 48%, and 41%, respectively. All cyclized analogues had similar activity compared to the wild-type TxIB, and the potency of them was not significantly different from that of TxIB. Thus, the cyclization modification of TxIB does not impact the activity of native α-conotoxin TxIB remarkably.

### 2.3. Serum Stability of Native TxIB and Its Cyclic Analogues 

**Figure 5:**
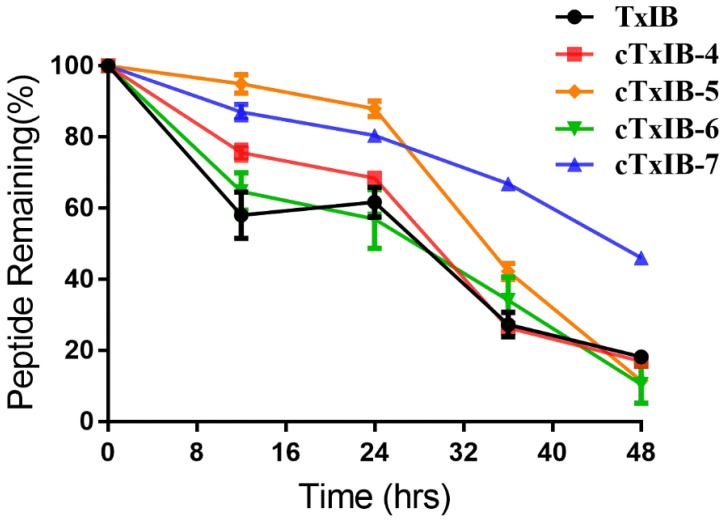
The relative stability of native TxIB and cyclic analogues in human serum. Error bars represent the mean ± SEM (n = 3).

The stability of TxIB and its cyclic analogues was tested in human serum to determine the effects of the cyclization modification. All peptides were incubated in human serum over 48 h at 37 °C. The amount of degradation products was quantified by the peak area with absorption at 214 nm using RP-UPLC. Native TxIB was cleaved rapidly in the initial stage, and approximately 42% TxIB was degraded in the first 12 h. From 12 to 24 h, there was no significant change in the amount of the remaining sample. Within 24 to 36 h, it was reduced by 34%. After 48 h, less than 20% of the peptide remained. After 48 h, the remaining amounts of cTxIB-4, 5, and 6 were similar to that of TxIB, indicating that their stability was slightly lower than that of TxIB. However, approximately 50% of cTxIB-7 remained after incubation in serum for 48 h, proving that it had significantly better stability than TxIB (Figure 5).

## 3. Discussion

α6β2* nAChRs regulate the release of dopamine and are important targets associated with a few neuropsychiatric diseases, including Parkinson’s disease and nicotine addiction [6]. The α-conotoxin TxIB selectively blocks α6β2* nAChRs while having no remarkable effect on other subtypes [18]. Consequently, TxIB can be used as a probe for studying α6β2* nAChRs, Parkinson’s disease, and nicotine addiction.

Biopharmaceutical drugs, including proteins and peptides, have gained much interest because of their high specificity, potency, and activity, and less toxicity compared to small molecules [40,41]. However, most peptides are easily degraded by proteases in vivo, resulting in low bioavailability, absorptivity, and short circulatory half-life [42,43]. Therefore, it is valuable to improve the bioavailability, stability, and absorption of peptide drugs by chemical modifications, such as cyclization, disulfide bond engineering, residue substitutions, N-terminal acetylation, glycosylation, PEGylation, etc. [23,24,25,26,27,28,29]. Cyclization of conopeptides has been proven as an effective strategy to stabilize the structure of peptides and protect against endopeptidases. There are many naturally occurring disulfide-rich macrocyclic peptides from animals, plants, and bacteria with exceptional stability [44]. The strategy to cyclize conopeptides artificially was originally inspired by these natural molecules. Besides, cyclization improved the protease resistance of several other peptide toxins, such as the scorpion toxin chlorotoxin and the sea anemone toxin APETx2 [45,46]. Here, the head-to-tail cyclization method was recruited to modify TxIB to improve the stability and maintain the potency of α6β2* nAChRs. 

Previous studies have demonstrated that linkers of appropriate size are important to retain the original structure and biological activity of wild-type peptides. Hence, we chose four, five, six, or seven amino acid residues to access the proper length of the linker based on the distance between N- and C-termini. Native TxIB and four linker variants were tested on rat α6β2* nAChRs at a concentration of 100 nM. All cyclic analogues retained a similar potency compared to TxIB. The results demonstrated that four linkers had no remarkable effect on the electrophysiological activity of TxIB. In addition, they also indicated that the modification of TxIB by head-to-tail cyclization would not affect its biological activity. 

To illustrate the stability of native TxIB and its cyclic analogues, they were incubated in male AB human serum at 37 °C. cTxIB-4, cTxIB-5, and cTxIB-6 had similar proportions of the original peptides remaining after 48 h in serum. On the contrary, about 50% of cTxIB-7 remained after 48 h, signifying that there was a tremendous improvement in the stability of the cyclized mutant using a linker with seven amino acid residues due to an increase of the structural rigidity of TxIB. The results indicated that the length of the linkers would affect the stability of cyclic peptides obviously, so the length of conotoxins should be considered according to their structure when they are ready to be cyclized. 

A variety of conotoxins have been cyclized in the past (Table 2). Among them, α-Ctx MII was the first conotoxin to be cyclized. The study indicated that the activity of cyclic MII mutants was preserved with linkers of six or seven amino acids. Meanwhile, the stability of them improved in human plasma [33]. Cyclic α-Ctx Vc1.1 with a six-residue linker had analgesic activity in a neuropathic pain model through oral administration [35]. For α-Ctx RgIA, its cyclic variant with a seven-residue linker inhibited α9α10 nAChRs with similar potency. Moreover, the six-residue and seven-residue linker mutants were the most stable in human serum [36]. Similar results were shown in this work compared to the above three conotoxins. These conotoxins contain some common characteristics in that they are globular disulfide isomers and hold highly constrained structures, so the cyclic analogues with long linkers have greater flexibility to reduce changes in the structure and hold their activity.

By contrast, several conotoxins with short linkers also improved the stability in serum or had a high resistance to enzymatic degradation, including ImI, AuIB, MrIA, and GeXIVA (Table 2). Among them, cyclic MrIA and GeXIVA with a linker of two amino acids hold similar activity and structure compared with the wild-type peptides. All cyclic AuIB analogues lost or reduced their activity at α3β4 nAChRs. Cyclic ImI with short linkers could resist trypsin degradation, but the activity of cyclic ImI was not tested. Together, the N-to-C-terminal backbone cyclization is an effective approach that stabilizes conotoxins to enhance their pharmaceutical potential, and the length of linkers depends on the structure of conopeptides.

In conclusion, the head-to-tail cyclization had no significant effect on the potency of α-conotoxin TxIB with a suitable linker at rα6β2* nAChRs and improved the stability in human serum. The results highlight the value of N- and C-termini cyclization for improving the biopharmaceutical properties of α-conotoxin TxIB and provide more useful support for the cyclization of other disulfide-rich peptides.

## 4. Materials and Methods 

### 4.1. Reagents and Materials

Human serum was purchased from Sigma (St. Louis, MO, USA). Dimethylformamide (DMF), dichloromethane (DCM), O-(7-azabenzotriazol-1-yl)-N,N,N′,N′-tetramethyluronium hexafluorophosphate (HATU), diisopropylethylamine (DIEA), triisopropylsilane (TIPS), and other chemicals for peptide synthesis were purchased from GL Biochem (Shanghai, China) and Applied Biosystems (Foster City, CA, USA). Acetonitrile (HPLC grade) was obtained from Fisher Scientific (Pittsburgh, PA, USA), and trifluoroacetic acid (TFA) from Tedia Company (Fairfield, OH, USA). Other reagents used were analytical grade. Reversed-phase HPLC preparative C18 Vydac column (10 μm, 22 mm × 250 mm) was obtained from Grace Vydac (Hesperia, CA, USA). ACQUITY UPLC BEH C18 Column (1.7 μm, 2.1 mm × 50 mm) was obtained from Waters (Milford, MA, USA).

### 4.2. Peptide Synthesis

Four linkers were designed to maintain the structure and native activity of TxIB, including GAAG, GGAAG, GAGAAG, and GGAAGAG. All peptides were synthesized using standard Fmoc solid-phase synthesis. TxIB was assembled on a Rink amide resin, while its analogues were prepared on an acid-sensitive resin 2-chlorotrityl chloride. To form two correct disulfide bonds, cysteine residues were protected in pairs with trityl (Trt) and acetamidomethyl (Acm). TxIB was deprotected and cleaved from the resin by treatment with a reagent K (trifluoroacetic acid/water/ethanedithiol/phenol/thioanisole; 90:5:2.5:7.5:5, *v*/*v*/*v*/*v*/*v*) for 2 h. The analogues were cleaved from the resin using a reagent (1% TFA in DCM, *v*/*v*) for 2 h. The released peptides were precipitated and washed three times with cold ether. Then, the analogues were cyclized in DMF with 5 mM HATU and 10 mM DIEA for 3 h and dried. The side-chain-protecting groups of the cyclic analogues except for acetamidomethyl (Acm) were removed by a solution (96% TFA/2% H_2_O/2% TIPS, *v*/*v*) for 1.5 h at room temperature. The released peptides were precipitated and washed three times with cold ether. 

All peptides were folded with a two-step oxidation protocol as described previously [18]. The first disulfide bond was formed in potassium ferricyanide buffer (20 mM K_3_[Fe(CN)_6_], 0.1 M Tris-HCl; pH 7.5) for 45 min. Then, the Acm groups were removed to form the second disulfide bond by iodine oxidation for 15 min. The two-step oxidation products were purified by RP-HPLC on a reversed-phase C18 Vydac column and the elution conditions were 5–40% buffer B within 45 min. Buffer A was 0.1% TFA in H_2_O and buffer B was 0.05% TFA in 90% acetonitrile. RP-UPLC was used to determine the purity of the peptides with absorption at 214 nm, and mass spectrometry was used to identify these products.

### 4.3. Electrophysiological Activity Measurements

The α6/α3 subunit is a chimera where the extracellular ligand-binding portion of the α6 subunit is spliced with the remaining α3 subunit [47]. This chimera was used to model the α6β2* ligand-binding domain because the injection of nonchimeric α6 with β2 fails to produce sufficient numbers of receptors [48]. The plasmids of rat α6/α3, β2 and β3 nAChR subunits were linearized by corresponding enzymes for in vitro cRNA transcription using the mMessage mMachine kit (Ambion, Austin, TX, USA). The cRNA was purified using the MEGA Clear Kit (Ambion), and then the 60 nL of purified cRNA was injected into oocytes with a Drummond microdispenser (USA). The oocytes were incubated at 17 °C with antibiotics during culture to prevent infection. After injection, the voltage-clamp recordings were performed after culture for another 4 days. The oocyte chamber consisting of a cylindrical well (50 μL in volume) was gravity-perfused at a rate of 2 mL/min with ND-96 solution (96.0 mM NaCl, 1.0 mM MgCl_2_, 2.0 mM KCl, 1.8 mM CaCl2, 5mM HEPES, pH 7.1–7.5) containing 1 μM atropine and 0.1 mg/mL bovine serum albumin. The oocyte was subjected once a minute to a 1-s pulse of 100 μM ACh for α6/α3β2β3 nAChRs. When a stable baseline was obtained, either ND-96 alone or ND-96 containing varying different concentrations of the conotoxins was pre-applied for 5 min before the addition of the ACh and recorded at room temperature (22 °C). 

### 4.4. Stability Assays

Serum stability of TxIB and cyclized TxIB analogues were carried out in male AB human serum. The serum was centrifuged at 14,000 g for 15 min to remove the lipids, and the supernatant was incubated for 15 min at 37 °C before the assay. Triplicate peptide samples were dissolved in human serum at a concentration of 100 μM and incubated at temperature of 37 °C. Then, the aliquots of each peptide were taken out at 0, 12, 24, 36, and 48 h. Each aliquot was quenched with 6 M urea and incubated for 10 min at 4 °C. Then, 20% TFA was used to precipitate serum proteins for an extra 10 min at 4 °C. All aliquots were centrifuged at 14,000 g for 15 min, and the supernatant was analyzed by RP-UPLC using a linear gradient of 5–30% buffer B for 5 min. Buffer A was 0.1% TFA in water and buffer B was 0.05% TFA in 90% acetonitrile. The remaining peptides were quantified by the peak area.

## Figures and Tables

**Table 1:** Theoretical and observed molecular weights of critical intermediates and final products analogues. MS profiles of the intermediates are given in Supplementary Material Figure S2.

Name	Theoretical Molecular Weight of Linear Peptide (Da)	Molecular Weight after Cyclization and Cleavage (Da)		Molecular Weight after Two-step Oxidation (Da)	
	Theoretical	Theoretical	Observed	Theoretical	Observed
cTxIB-4	1997.24	2125.24	2125.32	1979.24	1979.28
cTxIB-5	2054.30	2182.30	2182.23	2036.30	2036.22
cTxIB-6	2125.37	2253.37	2253.81	2107.37	2107.11
cTxIB-7	2182.42	2310.42	2310.45	2164.42	2164.32

**Table 2:** Summary of cyclic analogues of conotoxins.

Conotoxins	Sequences	Suitable Linkers	Targets	References
α-TxIB	GCCSDPPCRNKHPDLC*	GGAAGAG	α6/α3β2β3	This study
α-MII	GCCSNPVCHLEHSNLC*	GAGGAAG	α3β2	[33]
α-Vc1.1	GCCSDPRCNYDHPEIC*	GGAAGG	α9α10	[35]
α-RgIA	GCCSDPRCRYRCR	GGAAGAG/ GGAAGG	α9α10	[36]
α-ImI	GCCSDPRCAWRC*	A/AG	α3β2	[34]
α-AuIB	GCCSYPPCFATNPDC*	AG/AGGG/GGAA	α3β4	[37,39]
χ-MrIA	NGVCCGYKLCHOCAG	AG	NET	[32]
αO-GeXIVA	TCRSSGRYCRSPYDRRRR YCRRITDACV	GG	α9α10	[38]

## Supplementary Materials

The following are available online at https://www.mdpi.com/1660-3397/18/4/180/s1, Figure S1: RP-UPLC profiles of the cyclic products. The red asterisk indicated the target peak. (a) reaction time 3 h of cTxIB-6; (b) reaction time 6 h of cTxIB-6; (c) reaction time 3 h of cTxIB-7;(d) reaction time 6 h of cTxIB-7., Figure S2: Mass spectrometry analysis of the intermediates of cTxIB-4, 5, 6 and 7. (a) ESI-MS profile of cTxIB-4 with a mass of 2125.32 Da; (b) ESI-MS profile of cTxIB-5 with a mass of 2182.23 Da; (c) ESI-MS profile of cTxIB-6 with a mass of 2253.81 Da; (d) ESI-MS profile of cTxIB-7 with a mass of 2310.45 Da., Figure S3: RP-UPLC chromatograms of blank control and cTxIB-7. (a) blank serum; (b) a serum sample collected at 12 h after incubation of cTxIB-7 in serum; (c) a serum sample collected at 48 h after incubation of cTxIB-7 in serum.
