# Supplementary material for: Effects of Cyclization on Activity and Stability of α-Conotoxin TxIB
Source: Mar Drugs. 2020 Mar 29;18(4):180. doi: 10.3390/md18040180 (PMC7230940; doi:10.3390/md18040180)
Supplement: Supplementary file 1 [file marinedrugs-18-00180-s001.pdf]

Supporting Information

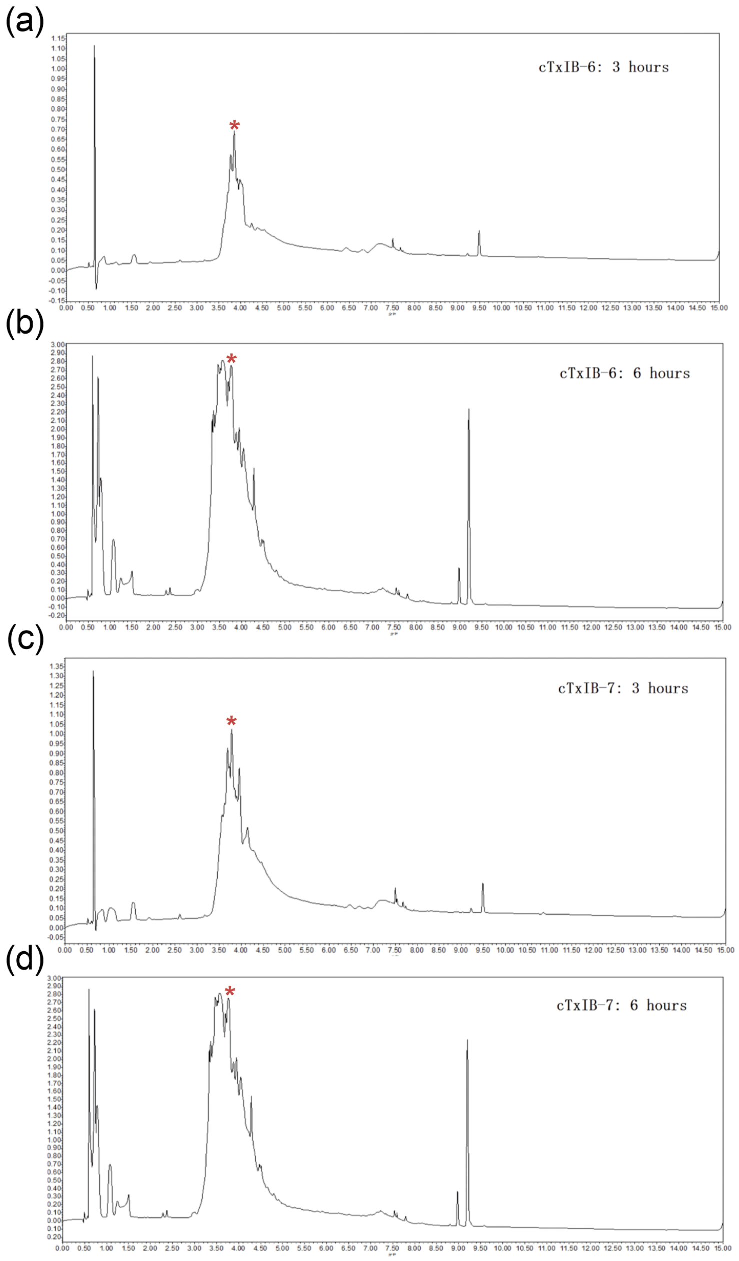

Figure S1. RP-UPLC profiles of the cyclic products. The red asterisk indicated the target peak. (a) reaction time 3 h of cTxIB-6; (b) reaction time 6 h of cTxIB-6; (c) reaction time 3 h of cTxIB-7;(d) reaction time 6 h of cTxIB-7.

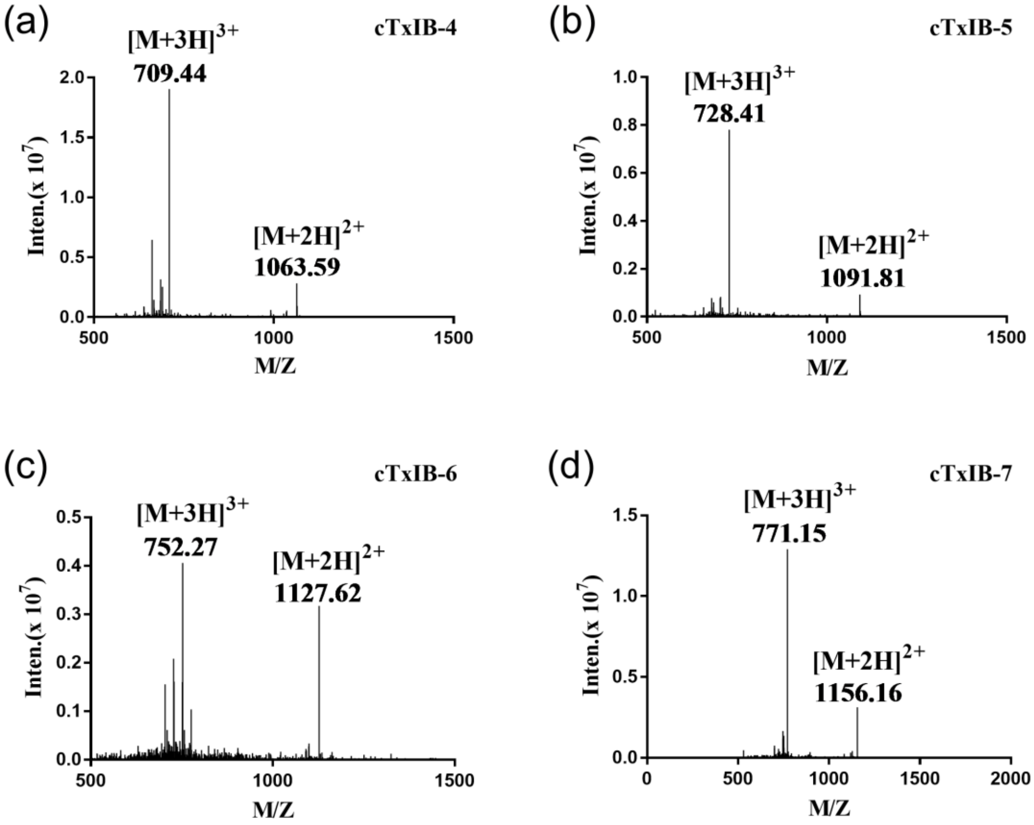

Figure S2. Mass spectrometry analysis of the intermediates of cTxIB-4, 5, 6 and 7. (a) ESI-MS profile of cTxIB-4 with a mass of 2125.32 Da; (b) ESI-MS profile of cTxIB-5 with a mass of 2182.23 Da. (c) ESI-MS profile of cTxIB-6 with a mass of 2253.81 Da. (d) ESI-MS profile of cTxIB-7 with a mass of 2310.45 Da.

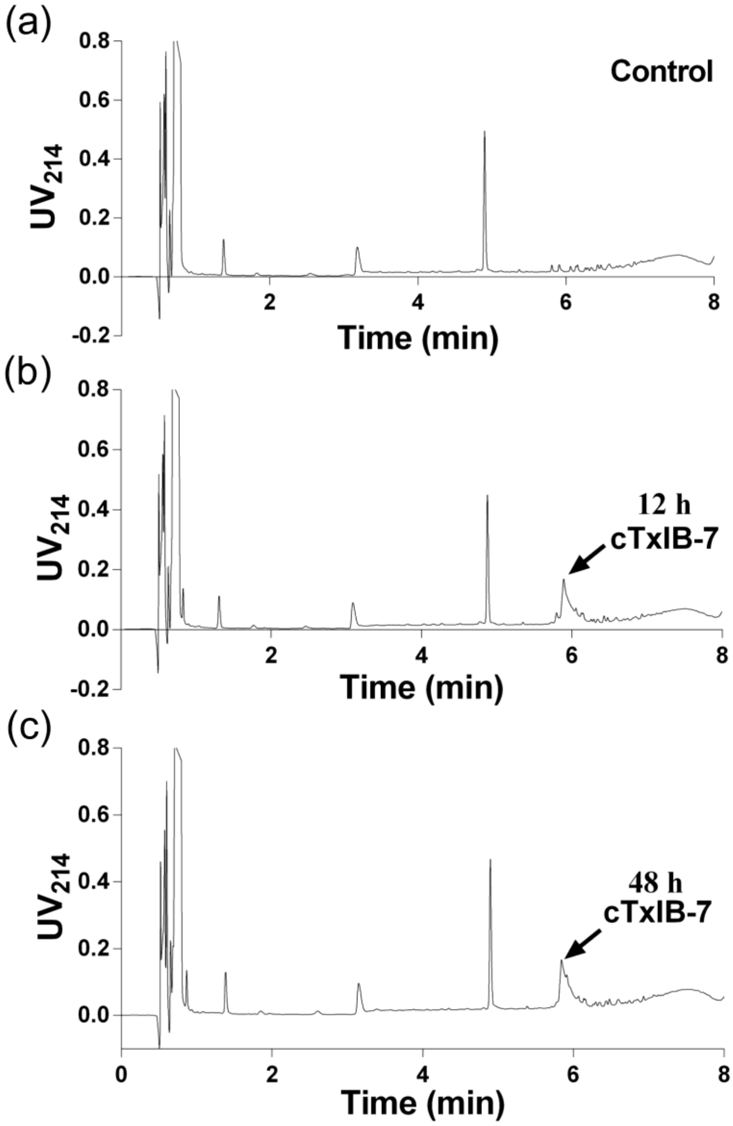

Figure S3. RP-UPLC chromatograms of blank control and cTxIB-7. (a) blank serum; (b) a serum sample collected at 12 h after incubation of cTxIB-7 in serum; (c) a serum sample collected at 48 h after incubation of cTxIB-7 in serum.
